# New Insights of Phospholipase A2 Associated Neurodegeneration Phenotype Based on the Long-Term Follow-Up of a Large Hungarian Family

**DOI:** 10.3389/fgene.2021.628904

**Published:** 2021-06-08

**Authors:** Renata Toth-Bencsik, Peter Balicza, Edina Timea Varga, Andras Lengyel, Gabor Rudas, Aniko Gal, Maria Judit Molnar

**Affiliations:** ^1^Institute of Genomic Medicine and Rare Disorders, Semmelweis University, Budapest, Hungary; ^2^Department of Neurology, Albert Szent-Györgyi Medical and Pharmaceutical Center, University of Szeged, Szeged, Hungary; ^3^MR Research Center, Semmelweis University, Budapest, Hungary

**Keywords:** neurodegeneration with brain iron accumulation II (NBIA2B), *PLA2G6* gene, PLA2G6-associated neurodegeneration (PLAN), neuroaxonal dystrophy, Dystonia-Parkinsonism syndrome

## Abstract

**Introduction:**

Phospholipase A2-associated Neurodegeneration (PLAN) is a group of neurodegenerative diseases associated with the alterations of PLA2G6. Some phenotype-genotype association are well known but there is no clear explanation why some cases can be classified into distinct subgroups, while others follow a continuous clinical spectrum.

**Methods:**

Long-term neurological, and psychiatric follow-up, neuropathological, radiological, and genetic examinations, were performed in three affected girls and their family.

**Results:**

Two 24-years old twins and their 22-years old sister harbored the p.P622S, and p.R600W mutation in PLA2G6. The age of onset and the most prominent presenting symptoms (gaze palsy, ataxia, dystonia, psychomotor regression indicated atypical neuroaxonal dystrophy (ANAD), however, optic atrophy, severe tetraparesis would fit into infantile neuroaxonal dystrophy (INAD). All siblings had hyperintensity in the globi pallidi and substantiae nigrae which is reported in ANAD, whereas it is considered a later neuroradiological marker in INAD. The slow progression, rigidity, bradykinesis, and the prominent psychiatric symptoms indicate PLA2G6-related dystonia-parkinsonism. Abnormal mitochondria, lipid accumulation and axonal spheroids were observed in the muscle and nerve tissue. Brain deposition appeared 6 years following the initial cerebellar atrophy. Mild MRI alterations were detected in the asymptomatic carrier parents.

**Conclusion:**

The colorful clinical symptoms, the slightly discordant phenotype, and the neuroimaging data in the family supports the view that despite the distinct definition of age-related phenotypes in PLAN, these are not strict disease categories, but rather a continuous phenotypic spectrum. The mild MRI alterations of the parents and the family history suggest that even heterozygous pathogenic variants might be associated with clinical symptoms, although systematic study is needed to prove this.

## Introduction

Phospholipase A2-associated Neurodegeneration (PLAN) (OMIM: 610217) is a group of neurodegenerative diseases that are associated with the alterations of the *PLA2G6* gene, resulting in pathological brain iron deposition (NBIA). PLAN is divided into four subgroups based on the phenotypes, age of onset and rate of progression: (1) classical infantile neuroaxonal dystrophy (INAD), (2) atypical neuroaxonal dystrophy (ANAD), (3) *PLA2G6*-related dystonia-parkinsonism (DP), and (4) autosomal recessive early onset parkinsonism (AREP) (Guo et al., 2018).

The PLA2G6 deficiency results in cerebellar atrophy and iron deposition in the basal ganglia and optic nerves (Carrilho et al., 2008). The neuropathological hallmark of the disease beside the iron accumulation, are the presence of neuroaxonal spheroids, abnormal α-synuclein proteins, hyperphosphorylated tau proteins, in some cases Lewy bodies (LBs), neurofibrillary tangles and neuropil threads (Guo et al., 2018).

The age of onset and the clinical manifestations are the main criteria used to make distinctions between the different subtypes of PLAN (Kurian et al., 2008); however, a broad and overlapping presentation of clinical phenotypes exist among the different subgroups (Ji et al., 2019). There are only a handful of publications with long-term longitudinal retrospective study designs, which help us to understand the transition from one subgroup to the other. Here, we present the long-term clinical retrospective study, the neuropathological and genetic features of a large family having one previously unreported and one known pathogenic compound heterozygous rare variants of the *PLA2G6* gene.

## Materials and Methods

### Molecular Genetic Analysis of the *PLA2G6* Gene

DNA samples were extracted from blood by the QIAamp DNA blood kit (Qiagen, Hilden, Germany), according to the manufacturer’s instructions. The total coding region of *PLA2G6* gene was genotyped with Sanger sequencing using both forward and reverse primers according to ABI Prism 3500 DNA Sequencer (Applied Biosystems, Foster City, United States). The sequences were aligned with the human reference genome (ENST00000332509.7, NM_003560) using BLAST application^1^.

### Multiplex Ligation-Dependent Probe Amplification

To identify exonic deletions and duplications within the *PLA2G6* of patients, multiplex ligation-dependent probe amplification (MLPA) was performed by the SALSA MLPA P120 PANK2/PLA2G6 (MRC Holland, Amsterdam, Netherlands), according to the manufacturer’s instructions. MLPA peak plots were assessed using COFFALYSER Software version (MRC Holland, Amsterdam, Netherlands).

### *In silico* Analysis

Mutation interpretation analysis was conducted using Alamut 2.0 (Interactive Biosoftware, San Diego, CA, United States). The significance of identified alterations was screened with HGMD^2^, dbSNP^3^, and ClinVar^4^ database. The novel alterations were established according to the American College of Medical Genetics and Genomics (ACMG) guidelines (Richards et al., 2015).

The results were analyzed with *in silico* prediction software **Polyphen2** (Adzhubei et al., 2010) (Polymorphism Phenotyping version 2)^5^, **SIFT** (‘‘Sorting Tolerant From Intolerant’’)^6^ (Kumar et al., 2009), and **Mutation Taster**^7^ (Schwarz et al., 2014).

### Histological Investigation (Muscle and Nerve Biopsy)

To confirm the diagnosis of PLAN, the IV/2 proband had muscle and sural nerve biopsy to confirm the presence of PLAN pathological hallmarks. Fresh frozen sections using the routine histochemical staining and araldite embedded ultrathin sections were prepared for light end electron microscopic examinations.

#### Case Report

Here, we are reporting a Hungarian family with three affected girls out of five children. This is a retrospective case study with institutional ethical committee approval. Informed consent for diagnostic genetic testing was obtained from each individual.

#### Family History

**FIGURE 1:**
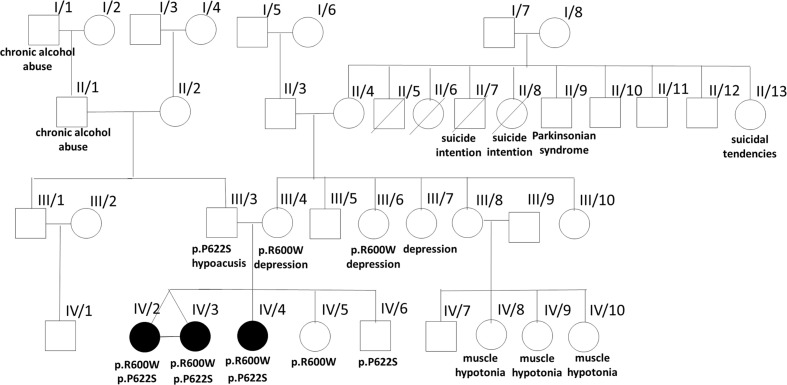
Pedigree of the affected family. The pedigree of the investigated family is presented with some clinical data from the family history and the results of the identified two *PLA2G6* gene mutations.

The mother of the proband (III/4) and two of her sisters (III/6 and III/7) were treated with depression. The maternal grandmother had a history of a benign renal tumor and cardiovascular diseases (II/4), in addition to the death of two of her siblings during early childhood due to unidentified causes. One of the aunts and uncle committed suicide (II/7 and II/8), another aunt had suicidal tendencies (II/13), while an uncle had late onset Parkinsonian syndrome (II/9). The healthy sister of the mother has four children, three daughters of which developed transient muscle hypotonia before the age of 2 years (IV/8, IV/9, and IV/10). Her son is healthy (IV/7). The father of the patient had hypoacusis and impulse control disorder (III/3), his father (I/1) and grandfather (II/1) were alcoholics (Figure 1).

## Results

### Patients’ History (Figure 2)

#### Patient IV/2 and IV/3 (24-Years Old)

Patient IV/2 and IV/3 were born as identical twins at the 38th gestational week with Apgar 10/10, followed by a few days of neonatal jaundice. They started to speak fluently at age of 2.5. The first symptoms presented at age of 3 in both children. The earliest signs were gait instability and speech deterioration, followed by the development of severe spastic paraparesis with moderate ataxia within a few years. At age of 14, IV/2 had mild dyskinesia and severe dystonia in the limbs. She became wheelchair-bound. At age 20 she had severe impulse control problems. At age 21, the dyskinesia progressed in the upper limbs, choreiform movements appeared and she became bradykinetic, the dysphagia and the ability to speak worsened and she had a generalized epileptic seizure. From age 22, she had episodic agitation, anxiety and acoustic hallucinations which were controlled by low dose quetiapine. One year later she lost her initiative and was characterized by amotivation, alogia, and apathy. The dysphagia, rigidity and bradykinesia were progressed.

**FIGURE 2:**
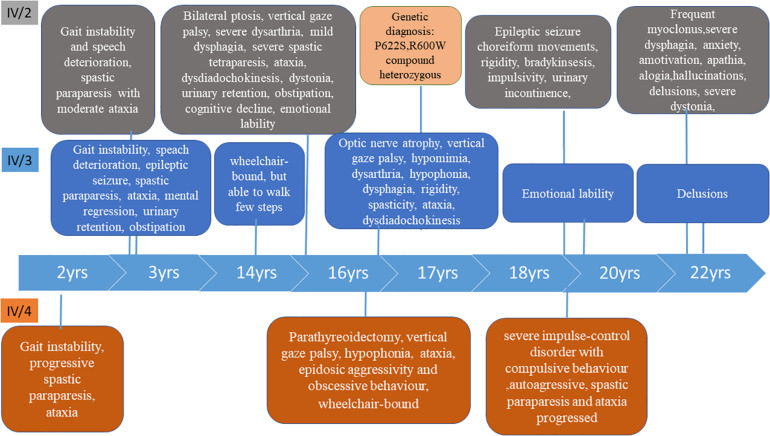
Timeline of the clinical symptoms in the three patients.

Neurological examination of patient IV/2 (at age 16) revealed macrocephaly, micrognathia, prominent forehead, bilateral ptosis, vertical gaze palsy, severe dysarthria, mild dysphagia, severe spastic tetraparesis in all limbs truncal and limb ataxia, upper limb dysdiadochokinesis, dystonia in the feet, hyperactive tendon reflexes with bilateral pyramidal signs, urinary retention and obstipation. She was able to stand with bilateral support. She developed severe cognitive decline and emotional lability during her follow-up. At age 21, she developed severe dyskinesia in both arms. Her speech regressed. She was able to communicate only with a few words.

Routine laboratory results, acylcarnitine profile, ceruloplasmin, ferritin, organic and amino acid levels were normal. Her EEG (electroencephalogram) detected bilateral synchronic theta bursts and irritative signs in the frontal regions. ENG (electronystagmography) revealed axonal neuropathy. EMG (electromyography) was normal at age 10 years. BAEP (brainstem auditory evoked potential) detected bilaterally increased wave V. latency. VEP (Visual Evoked Potential) detected bilateral prolonged P100 latency. The abdominal ultrasound was normal.

Patient IV/3 was 3-years old at the time of developing the first psychiatric symptoms (irritability and emotional lability) and the first epileptic seizure. A few years later she developed urinary retention and constipation. The disease progressed, her speech deteriorated quickly and severe somatomental regression was present at the age of 10. She became wheelchair-bound at age of 14. Her mood was stable. She did not have chorea, but dystonia has been observed in the feet. Although her spastic paraparesis and ataxia progressed, she is able to walk a few steps with aid at the age of 22. She has obsessive-compulsive symptoms.

Neurological examination of patient IV/3 at age 16 detected micrognathia, synophrys, bilateral optic nerve atrophy (without retinal hyperpigmentation), supranuclear vertical gaze palsy, hypomimia, mild dysarthria, hypophonia, dysphagia. Rigidity was observed in the arms, spasticity in the legs. She had proximal tetraparesis, severe truncal and limb ataxia, dysdiadochokinesis in the upper limbs, hyperactive tendon reflexes with bilateral pyramidal signs and urinary incontinence. She was able to walk with aid. Mental regression, obsessive compulsive behavior and anxiety were detected. At age 18, she developed dystonia in the feet. Her disease progressed very slowly. At age 22, she is still able to walk with bilateral support. Her speech did not deteriorate.

She had slightly increased serum cholesterol and decreased triglycerides. Other laboratory test results were within the normal range. Because of the supranuclear vertical gaze palsy and psychiatric signs associating with movement disorder bone marrow aspiration was performed, in which sea blue histiocytes were present. EEG showed intermittent generalized slow and fast paroxysms EMG, ENG, and BAEP were normal at age 10 years. VEP detected bilateral prolonged P100 latency at age 16.

#### Patient IV/4 (22-Years Old)

She was born at week 40 with Apgar 10/10. She had normal postnatal somatomental development, started to walk at the age of 10 months and talked at 14 months. The first signs presented at the age of 2 with gait instability. Progressive spastic paraparesis developed with ataxic gait. She had parathyroidectomy and subtotal thyroidectomy due to a parathyroid adenoma at the age of 14. At age 15, she was not able to stand up alone or to walk without support; at age 20 she became wheelchair-bound. At age 14, she was episodically aggressive and an obsessive behavior started, which worsened at age 18, at the time of which she became autoagressive.

Neurological examination of the patient at age 14 revealed vertical gaze palsy, hypophonia, and severe spastic tetraparesis with proximal predominance. Both hands and feet were in a dystonic posture. She had truncal and limb ataxia, hyperactive tendon reflexes with pyramidal signs and urinary incontinence. A severe cognitive deficiency was present. At age 18, she developed severe impulse-control disorder with compulsive behavior and autoaggressiveness. At age 20, her spastic paraparesis and ataxia progressed.

She had increased serum cholesterol and triglyceride. Other routine laboratory results, acylcarnitine profile, organic acid, and amino acid levels were normal. Filipin staining was stronger in her fibroblasts than in the normal control, but the level of oxysterol was normal. The abdominal ultrasound detected nephrolithiasis. EMG and ENG were normal at age 8 years, two years later mild axonal lesion was detected. EEG registered diffuse background slowing with bursts of generalized slow wave paroxysms and fast rhythms. BAEP was normal. VEP detected prolonged P100 latency on both side.

### Neuroimaging

**FIGURE 3:**
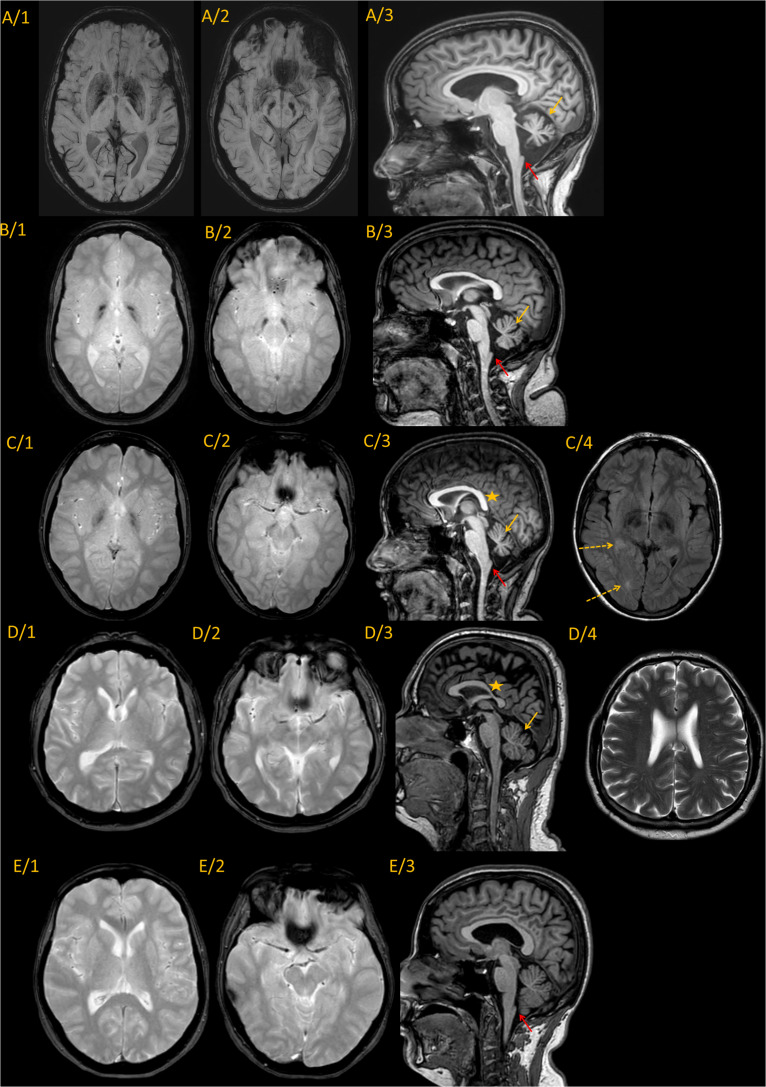
Representative MRI imaging pictures of the family. Every line represents images from a single individual. **(A)** IV/2; **(B)** IV/3; **(C)** IV/4; **(D)** III/3; and **(E)** III/4 Column 1–2. Axial iron sensitive sequences (SWI in line A, and T2* in the remaining) through the basal ganglia and midbrain. Column 3: Sagittal midline T1 images. Findings are summarized in the associated table. Every patient diagnosed with PLAN **(A–C)** showed iron accumulation in the globi pallidi internus and substantiae nigrae, while the carrier parents did not show iron accumulation. Cerebellar atrophy was present in all the three affected children [**(A–C)**, yellow arrows], and was also present at the father [**(D/3)**, yellow arrow]. The characteristic vertical splenium corpus callosum was present at patient IV/4 [**(C/3)**, yellow star], however, also at the father corpus callosum was atypical [markedly thin isthmus in an adult; **(D/3)** yellow star]. The apparent claval hypertrophy sign was present at all three affected children [**(A–C)**, red arrow], and was identifiable at also the carrier mother [**(E/3)**, red arrow]. Besides, patent IV/4 also had white matter lesions identifiable on T2 images [**(C/4)**, dotted arrows]. At the father enlarged vascular spaces were detected **(D/4)**. In summary, it seems, that besides the characteristic PLAN-associated signs at the affected children, both parents showed mild abnormalities, which might be related to the PLA2G6 carrier state, without pathological iron accumulation.

The MRI was followed up from age 10 and 8 years in the sisters. The first MRI detected cerebellar atrophy and mild cerebral atrophy in patient IV/2. In patient IV/3 at age 10 only mild cerebral atrophy was present. Patient IV/4 had mild periventricular T2 hyperintensity. The brain iron accumulation was identified 6 years later in all three children. The last representative brain MRI images of the patients and their parents are shown in Figure 3, and the detected abnormalities are summarized in Table 1. All three affected children demonstrated brain iron accumulation in the the globi pallidi internus (Gpi), and substantiae nigrae, while this was absent in the parents. Cerebellar atrophy and the so-called apparent claval hypertrophy was a consistent feature in all three affected children, and patient IV/4 presented the characteristic sign of vertically oriented splenium of the corpus callosum, as well as white matter lesions. The parents also showed mild MRI abnormalities without brain iron accumulation, which may be related to the *PLA2G6* carrier state. The father had mild cerebellar atrophy and the thinning of the isthmus of the corpus callosum and enlarged vascular spaces. In the mother, the apparent claval hypertrophy sign was present. Patient IV/2 had a DaTscan which displayed normal levels in the right basal ganglia and decreased DaT uptake on the left side.

**TABLE 1:**
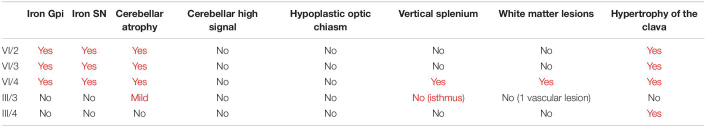
The results of the brain MRI.

*The identified morphological alterations detected by the brain MRI in the patients and their parents.*

### Histological Investigation (Muscle and Nerve)

**FIGURE 4:**
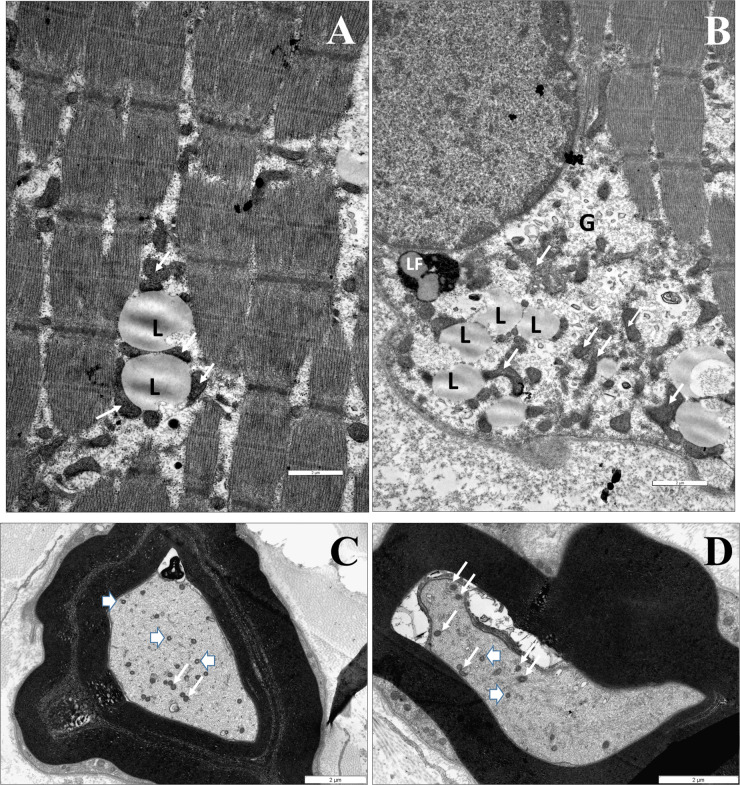
The identified neuropathological alterations. Muscle biopsy of IV/2 proband **(A,B)**. **(A)** EM shows an accumulation of particular-shaped mitochondria and lipid vacuoles in the intermyofibrillar space (L: lipid vacuole, white arrow: mitochondrium). **(B)** The accumulation of lipid vacuoles and glycogen with slight mithocondrial proliferation in the subsarcolemmal area (L: lipid vacuole, LF: lipofuscin, G: glycogen, and white arrow: mitochondrium). Nerve biopsy of IV/2 proband **(C,D)**: **(C)** Slightly enlarged mitochondria an axon (white arrows: mitochondrion). The myelin is decompacted, **(D)** EM shows dystrophic axons: Tubulovesicular structures can be observed in the spheroid, as well as degenerated vesicles and vacuoles. Scale bars: 2 μm (arrowhead: spheroid body).

In the muscle biopsy of patient IV/2 dominantly scattered rounded atrophic muscle fibers and rarely occurring angular atrophic fibers were observed. EM detected accumulation of lipid vacuoles and glycogen with slight mitochondrial proliferation in the subsarcolemmal and intermyofibrillar area. At age 16 the number of axons in the sural nerve was reduced by 30%, with the presence of small regenerating axon clusters. Some of these axons had a thinned myelin sheath. Electron microscopy detected decompactation of myelin, tubulovesicular structures in the spheroids, and enlarged, abnormal mitochondria (Figures 4A–D).

### Genetic Investigations

In the *PLA2G6* gene a novel p.P622S (c.1864C > T) and a known pathogenic p.R600W (c. 1798 C > T) rare variants were present in all siblings. The novel p.P622S variant (prediction scores: GERP: 4.43, Mutation Taster: 0.9875 – disease causing, Polyphen-2_HVAR (0.297), SIFT: 0.453 – tolerated) was classified as variant of uncertain significance according to ACMG guideline (PM1, PM2, PP2, and BP4) (Richards et al., 2015). Segregation analysis detected the heterozygous P622S mutation in the father of the proband, while, the heterozygous R600W mutations was identified in the mothers. One of their healthy siblings inherited the PLA2G6 genotype of the mother, the other the genotype of the father. The aunt III/6 harbored the R600W mutation too. The sequence of the *NPC1 (NM_000271.5), PANK2 (NM_153638.3), MPAN (NM_031448.6)*, and *CP (NM_000096.4)* genes were normal. Large deletions and/or duplications of the PLA2G6/PANK2 gene were excluded by Multiplex Ligation-Dependent Probe Amplification (SALSA MLPA probe mix P120B2; MRC Holland, Amsterdam, Netherlands).

## Discussion

Here, we report a long-term follow-up study of a Caucasian family with three affected children harboring one novel and one known pathogenic mutations of *PLA2G6* gene leading to a colorful clinical picture. Several authors have highlighted the heterogeneity of the clinical and radiological presentation in PLAN associated disorders (Nardocci et al., 1999; Gregory et al., 2008, 2009; Karkheiran et al., 2015). The studied family further demonstrates that the disease is indeed a spectrum rather than discrete entities. The disease onset and the most prominent features, such as eye movement disturbances, ataxia, dystonia, psychomotor regression, and cerebellar atrophy with iron accumulation and the relatively slow progression best resemble ANAD (Table 2), however, some features are more closely related to INAD or Dystonia-Parkinsonism. For example the severe tetraparesis and the detected MRI changes (prominent cerebellar atrophy with the apparent claval hypertrophy and corpus callosum alterations) are more characteristic of INAD. The optic nerve atrophy observed in one twin also supports this notion (Gregory et al., 2008). The finding of MRI hyperintensity in both the globus pallidus and substantia nigra is reported in children having ANAD, whereas it is considered a later neuroradiological marker in INAD. On the other hand, the predominant neuropsychiatric features, Parkinsonism in conjunction with decreased DaT signal are more characteristic of the Dystonia-Parkinsonism phenotype (Gregory et al., 2009). All children had OCD symptoms, displayed most prominently in the youngest girl, who had severe anxiety and autoagression, associated with severe impulsivity. One of the twins also had severe agitation, hallucinations and severe anxiety. None of the studied individuals had depression. While the severe speech deterioration is not reported in the literature, it can be a component of the severe psychomotor regression. We also emphasize the prominent expression of minor abnormalities (macrocephaly, micrognathia, prominent forehead, and synophris) in all affected members of the family, which are not frequently mentioned in the literature.

**TABLE 2:**
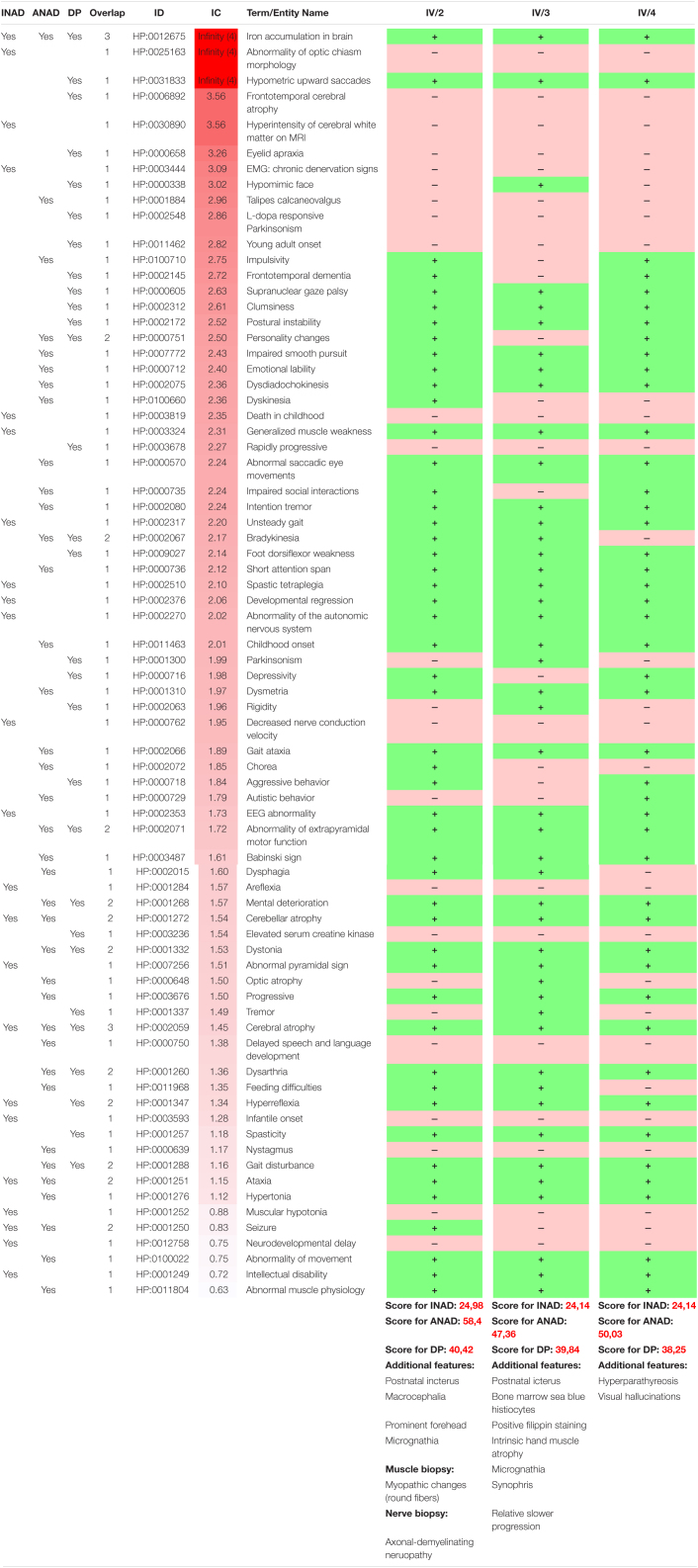
The phenotypic overlap of the patients with the allelic disorders associated with *PLA2G6.*

*The table shows the phenotypic overlap of the three affected individual with the allelic disorders associated with PLA2G6 gene. Phenotypic features were extracted from the OMIM clinical synopsis table, then mapped to their corresponding human phenotype ontology (HPO) term, and sorted according to their specificity (i.e., information content = IC). In the first three column we “yes” designates, that the HPO-term in the given row is associated with the allelic disorder in the OMIM. In the last three column “+” or “−” designates whether the given phenotypic abnormality is present in the given family member or not. Scores for the allelic disorders (INAD, ANAD, and DP) were calculated for the patients as following: if the given patient showed a symptom, which is present in a given allelic disorder, then she received a score for that allelic disorder equal to the information content of that phenotypic abnormality. All the phenotypic abnormality were then summed for every patient and every allelic disorder. Additional features of the patients, which are not described in OMIM, but are considered important are listed for every patient.*

In PLAN, the neuroradiological features are changing dynamically. The first sign in the siblings in our study was cerebellar atrophy without brain iron accumulation. This is in accordance with the observation of Darling et al. who concluded that cerebellar atrophy is a universal radiological sign in infantile and childhood onset PLAN and it is correlated with the severity of the phenotype. The presence of the iron accumulation within the globi pallidi and substantiae nigrae is also a common uniform feature regardless of the phenotype (Nardocci et al., 1999; Darling et al., 2019), however, iron deposition was not detected on initial neuroimaging in many of the infantile cases (Illingworth et al., 2014).

Upon consideration of the longitudinal history of our cases and the literature data, we can conclude that the clinical picture, the neuroradiological and electrophysiological parameters changed dynamically with the disease progression. Historically, the classification of the disease subgroups was based primarily on the age at onset and selected biomarkers. At this is not always feasible, use of the term PLAN spectrum disease is more appropriate (Ozes et al., 2017). Recently the spectrum was further expanded with complicated hereditary spastic paraparesis (Koh et al., 2019) and pure Parkinsonism without iron accumulation also (Chu et al., 2020). Intrafamilial discordant clinical phenotype such as spastic paraplegia and dopa responsive Parkinsonism with claval hypertrophy ad without brain iron accumulation was reported by Park et al. (2019). In this family, the patients had homozygous R747W mutations in PLA2G6. Here we report a new intrafamilial clinical discordancy in association with compound heterozygous *PLA2G6* mutations (p.R600W, p.P622S). Both of these missense variants are located in a mutational hot spot region found, in a critical and well-established functional PNPLA domain, which can explain the loss of function of the PLA2G6 protein. The p.R600W is a new disease causing mutation. Another amino acid change at the same position in the PLA2G6 gene (R600Q) has previously been reported in a homozygous state in a patient with INAD (Paisan-Ruiz et al., 2012).

The family had a long diagnostic journey since the vertical gaze palsy, the sea-blue histiocytes, and the positive filipin staining misled the clinical diagnostic. Since NPC1 and hematological disorders were excluded, sea blue histiocytes could be explained by enhanced creation of ceroid lipofuscin concentrating in macrophages. This can occur in NBIA as well, which is consistent with the consensus that defective lipid metabolism is present in various types of NBIA (Aoun and Tiranti, 2015; Colombelli et al., 2015). In vitro study of the *PLA2G6* knockouts detected neuropathological alterations similar to the ones described in our cases (Beck et al., 2011).

The family history of the siblings is also very colorful. Several symptoms potentially associated with PLAN are present from both paternal and maternal sides. Karkheiran et al. (2015) and Guo et al. (2018), respectively, have reported depression and Parkinsonism as a presenting symptom in several families. In this family both symptoms occurred frequently. Unfortunately, the segregation studies could not be performed in all relatives. Other authors have suggested that heterozygous PLA2G6 variants might be a risk factor for sporadic Parkinson’s disease (Gui et al., 2013; Daida et al., 2021). Daida et al. (2021) reported that the variants of PLA2G6 may be associated with both familial and early onset sporadic Parkinson’s disease either as a monogenic cause (harboring homozygous or compound heterozygous mutations) or as a genetic risk factor (harboring heterozygous mutation. In the family described by us, the parents until age of 57 did not show signs of Parkinsonism; however, the MRI detected mild abnormalities which might be in association with PLA2G6 variants. One maternal uncle had late onset PD. Our hypothesis is that the presence of the heterozygous pathogenic variants are not completely silent clinically, however, this must be studied systematically in a larger number of families.

In conclusion: the colorful clinical symptoms, the slightly discordant clinical picture in the identical twins and the neuroimaging data in the family support the view that despite the distinct age-related phenotypes defined in PLAN, strict disease categories cannot be established, rather, a continuous phenotypic spectrum with variable age at onset should be considered. The phenotype is not always in accordance with the classical disease subgroups, especially when findings are based solely on cross-sectional clinical data. The mild MRI alterations of the asymptomatic parents and the family history suggest that heterozygous pathogenic variants might be associated with clinical symptoms, although systematic study of heterozygous family members is warranted.

## Data Availability Statement

The datasets presented in this study can be found in online repositories. The names of the repository/repositories and accession number(s) can be found in the article/supplementary material.

## Ethics Statement

The studies involving human participants were reviewed and approved by The Hungarian Research Ethics Committee. Approval number was: 44599–2/2013/EKU (535/2013.) The patients/participants provided their written informed consent to participate in this study.

## Author Contributions

RT-B and MM designed and conducted the study. PB, EV, AL, and MM investigated the patients and their family members, and analyzed the clinical data. GR performed the MRI studies. RT-B and AG performed the genetic examinations. RT-B, PB, and MM wrote the manuscript. All authors read and approved the final version of the manuscript.

## Conflict of Interest

The authors declare that the research was conducted in the absence of any commercial or financial relationships that could be construed as a potential conflict of interest.

## Footnotes

1
https://blast.ncbi.nlm.nih.gov/Blast.cgi

2 www.hgmd.cf.ac.uk

3 www.ncbi.nlm.nih.gov/SNP/

4 www.ncbi.nlm.nih.gov/clinvar/

5 http://genetics.bwh.harvard.edu/pph2/

6 http://sift.jcvi.org/

7 http://www.mutationtaster.org/
